# Hantavirus Immunology of Rodent Reservoirs: Current Status and Future Directions

**DOI:** 10.3390/v6031317

**Published:** 2014-03-14

**Authors:** Tony Schountz, Joseph Prescott

**Affiliations:** 1 Arthropod-borne and Infectious Diseases Laboratory, Department of Microbiology, Immunology and Pathology, College of Veterinary Medicine, Colorado State University, Fort Collins, CO 80524, USA; 2 Laboratory of Virology, Division of Intramural Research, National Institute of Allergy and Infectious Diseases, National Institutes of Health, Rocky Mountain Laboratories, Hamilton, MT 59840, USA; E-Mail: prescottjb@niaid.nih.gov

**Keywords:** hantavirus, rodent, immune response, ecoimmunology, zoonosis, systems biology

## Abstract

Hantaviruses are hosted by rodents, insectivores and bats. Several rodent-borne hantaviruses cause two diseases that share many features in humans, hemorrhagic fever with renal syndrome in Eurasia or hantavirus cardiopulmonary syndrome in the Americas. It is thought that the immune response plays a significant contributory role in these diseases. However, in reservoir hosts that have been closely examined, little or no pathology occurs and infection is persistent despite evidence of adaptive immune responses. Because most hantavirus reservoirs are not model organisms, it is difficult to conduct meaningful experiments that might shed light on how the viruses evade sterilizing immune responses and why immunopathology does not occur. Despite these limitations, recent advances in instrumentation and bioinformatics will have a dramatic impact on understanding reservoir host responses to hantaviruses by employing a systems biology approach to identify important pathways that mediate virus/reservoir relationships.

## 1. Introduction

Hantaviruses (family *Bunyaviridae*, genus *Hantavirus*) are negative-stranded, trisegmented viruses that cause about 200,000 disease cases annually, with case fatality rates of 0.5%–40%, depending on the virus [1]. The viral gene segments encode four or five polypeptides. The large (L) segment encodes the RNA-dependent RNA polymerase (RdRp), the medium (M) segment encodes a precursor that is posttranslationally cleaved into Gn and Gc glycoproteins, and the small (S) segment encodes the nucleocapsid (N) protein. Some hantaviruses encode a second nonstructural polypeptide (NSs) downstream from the N start site in frame 2 [2,3]. Little is known about the immunomodulatory abilities of these proteins; however, there is evidence the N, Gn and NSs may alter host cellular responses during infection.

More than 20 hantavirus species have been classified, and many more unclassified hantaviruses have been identified. They are hosted by several species of rodents (Rodentia), insectivores (Insectivora), and bats (Chiroptera) [4]. Little work has been conducted to understand hantavirus infections of insectivores or bats; however, much is known about the ecology of rodent-borne hantaviruses because of their impact on human health (Table 1). A central problem of hantavirus/reservoir host research is the lack of reagents and methods for experimentally examining the immune response. Recent experimental work on the immunology of rodent reservoirs, summarized in an exceptional review by Easterbrook and Klein [5], has begun to clarify these issues. The immune response is energetically expensive for wild animals, thus the findings of experimental studies will be critical for understanding the ecoimmunology of reservoir hosts of hantaviruses [6,7], and experiments using wild rodents in natural or semi-natural environments [8,9] will be required to validate laboratory findings.

Rodentia is the largest mammalian order and is comprised of about 1,800 species [10]. Only a few dozen species have been identified as susceptible hosts, and most hantaviruses are hosted by a single rodent species [11]. In each hantavirus/rodent reservoir relationship, infection results in two prominent features: no conspicuous pathology and persistent infection [12,13,14]. The earliest report of experimental infection of a reservoir host with its hantavirus was by Lee *et al*. [15] that described infection of striped field mice (*Apodemus agrarius*) with Hantaan virus. Inoculated mice developed chronic infection with transient viremia, and shed virus principally in urine, saliva and, to a lesser extent, feces, despite the production of neutralizing antibodies.

Currently, three laboratory infection systems have been developed to study hantavirus infections of reservoir hosts: Seoul virus (SEOV) infection of the Norway rat (*Rattus norvegicus*), Puumala virus (PUUV) infection of the bank vole (*Myodes glareolus*), and Sin Nombre virus (SNV) infection of the deer mouse (*Peromyscus maniculatus*) [12,14,16]. In humans, these viruses are etiologic agents of hemorrhagic fever with renal syndrome (HFRS; SEOV, PUUV) and hantavirus cardiopulmonary syndrome (HCPS; SNV) [1]. These diseases share many pathologic similarities and because little damage to the endothelium occurs during infection, it is thought that the inflammatory immune response contributes to pathogenesis [17,18]. Because the Norway rat is a model organism with many specific reagents, including monoclonal antibodies to immune markers, significant progress has been made toward understanding the reservoir host immune response to SEOV [19,20,21]. Fewer methods and reagents are available for bank voles and deer mice. Despite these limitations, emerging technologies will be useful for understanding why rodent reservoirs are infected without disease and why they are unable to clear infection.

**Table 1:** Pathogenic hantaviruses and their principal reservoir hosts.

Virus	Reservoir	Disease *
Hantaan virus	*Apodemus agrarius*	HFRS
Dobrava virus	*Apodemus flavicollis*	HFRS
Seoul virus	*Rattus norvegicus*	HFRS
Saaremaa virus	*Apodemus agrarius*	HFRS
Amur virus	*Apodemus peninsulae*	HFRS
Puumala virus	*Myodes glareolus*	HFRS
Sin Nombre virus	*Peromyscus maniculatus*	HCPS
New York-1 virus	*Peromyscus leucopus*	HCPS
Monongahela virus	*Peromyscus leucopus*	HCPS
Bayou virus	*Otyzomys palustris*	HCPS
Black Creek Canal virus	*Sigmodon hispidis*	HCPS
Andes virus	*Oligoryzomys longicaudatus*	HCPS
Laguna Negra virus	*Calomys laucha*	HCPS
Araraquara virus	*Bolomys lasiurus*	HCPS
Choclo virus	*Oligoryzomys fulvenscens*	HCPS
Juquitiba virus	*Oligoryzomys nigripes*	HCPS
Castelo dos Sonhos	*Oligoryzomys utiaritensis*	HCPS
Bermejo virus	*Oligoryzomys chacoensis*	HCPS
Lechiguanas virus	*Oligoryzomys flavescens*	HCPS

* HFRS, hemorrhagic fever with renal syndrome; HCPS, hantavirus cardiopulmonary syndrome.

A significant limitation of HFRS research is that none of the HFRS-causing hantaviruses cause disease in animal models. However, two New World hantaviruses cause an HCPS-like disease in Syrian golden hamsters (*Mesocricetus auratus*): Andes virus (ANDV) and Maporal virus (MAPV) [22,23]. ANDV causes most HCPS cases in South America; however, no human cases of disease have been associated with MAPV. ANDV is hosted by the long-tailed pygmy rice rat (*Oligoryzomys longicaudatus*) and MAPV is hosted by the delicate pygmy rice rat (*Oligoryzomys delicatus*) [24,25]. ANDV is considered an animal biosafety level-4 pathogen in most nations, whereas MAPV is considered an ABSL-3 virus [26].

## 2. Infection of Rodent Reservoirs

The natural route of transmission among reservoir rodents is thought to be principally through aerosols and/or biting [27]. However, experiments have been equivocal in clarifying transmission mechanisms. Weanling bank voles caged with infected individuals lead to transmission as early as 14 days post exposure [14]. Similar experiments examining SNV transmission between deer mice have been less informative; however, in artificial enclosure experiments, transmission appeared to be facilitated by deer mice with higher amounts of viremia and wounding [8] and males likely play a dominant role in transmission in natural populations [9,28,29]. In bank voles, offspring of PUUV-infected dams were less likely to be infected after exiting the nest because of protective maternal antibody [30]. Although it is thought horizontal transmission is most important, work by Hutchinson *et al.* [31] showed that vertical transmission occurred among cotton rats (*Sigmodon hispidus*) infected with Black Creek Canal virus, so it is possible that both routes may influence transmission at the population level. 

The route of transmission has important ramifications in terms of the host immune response where, presumably, a mucosal response occurs with aerosol transmission and a localized response at a bite site. Experimental data have also shown that patterns of the expression of genes related to the immune response are different in infected males and females [32], and it is likely these differences have important roles in hantavirus ecology. Spillover to other rodent species also occurs [33,34,35,36], but it is unknown if the rodents remain infected. Recent work has shown that deer mice are experimentally susceptible to ANDV; however, virus is cleared several weeks after infection [37]. A pronounced Th2/Tfh gene expression profile occurs, including IL-4 pathway activation, that does not appear to be substantially activated in SNV-infected deer mice [13,38]. This system provides an opportunity to identify viral and reservoir host factors that are important for sterilizing immunity that clears infection.

The principal target cells of infection in rodents (and humans) are the microvasculature endothelial cells of many tissues [39]. Experimental intramuscular infection of deer mice with SNV resulted in detectable virus in the lungs as few as two days later [13]. Many organs appeared infected, although infection was limited to the vasculature within those tissues. Two infectious outcomes occur in experimentally infected deer mice; a *disseminated infection* of three or more organs, or a *restricted infection* of the lungs and heart [40]. The relevance of these two patterns to transmission efficiency is unknown. The levels of viral RNA vary dramatically in infected deer mice, with most having modest to moderate levels of RNA at the peak of infection. However, some deer mice have significantly greater amounts of viral RNA, suggesting these deer mice may produce substantially more virus than others, and it is possible they transmit virus more efficiently (e.g., “supershedders”) [13]. This also occurs in semi-natural transmission experiments [9] and suggests certain individuals may have a prominent role in population-level transmission of hantaviruses. 

## 3. Antibody Responses

Most serological assays for detecting antibody responses in hantavirus reservoirs use virus neutralization, ELISA or strip immunoblotting [12,29,41,42,43]. While some of these assays are IgG-specific, others use antiserum to whole IgG, including the light chains. Since light chains are shared by all immunoglobulins, these detection antibodies are not IgG-specific. Moreover, no assays are in place for detecting IgA, which should be prominent in mucosal infections. IgM assays have been problematic despite the availability of anti-IgM capture antisera that are cross-reactive with IgM from at least one hantavirus reservoir species [44]. Some immunoglobulins have isotypes with specific effector activities, such as complement fixation or antibody-dependent cell cytotoxicity. Laboratory house mice have four IgG isotypes; IgG1, IgG2a, IgG2b and IgG3. It is likely that reservoirs also have immunoglobulin isotypes with distinct effector functions and which might predominate during hantavirus infections. These reagent deficiencies are a current obstacle for assessing antibody responses in rodent reservoir hosts. Despite these limitations, many field studies have been conducted examining antibody responses in natural and semi-natural hantavirus infections of rodent reservoirs [34,45,46,47,48,49,50,51]. 

In experimentally-infected deer mice, SNV nucleocapsid-specific antibodies can be detected in serum as early as 10 days post infection, and neutralizing antibody can be detected after three weeks post infection [13]. Similarly, experimentally-infected bank voles produce PUUV-specific antibodies two to three weeks after inoculation [14] and rats experimentally infected with SEOV also produce IgG within two weeks post inoculation [52]. The presence of IgG in these naturally and experimentally infected reservoirs is an indicator of class switching and affinity maturation, events that are mediated by T cells [53]. Thus, rodents mount adaptive T cell/B cell immune responses to their reservoir hantaviruses; however, it appears to be inadequate for virus clearance. While inflammatory signatures are present [13,20,54], the magnitude of these signals appears to be modest relative to expression levels found in a Syrian hamster pathology model of HCPS [55]. It is noteworthy that immunization of rodent reservoirs with homologous nucleocapsid antigen or plasmids encoding the antigen protects from subsequent challenge [56,57], suggesting infection can be prevented in reservoir hosts.

## 4. Signatures of Immunomodulatory Activities of Hantaviruses

Several hantavirus proteins have been implicated in modulation of the host cell’s antiviral defenses (Table 2). The Gn glycoproteins of pathogenic New World hantaviruses and SEOV possess an immunoreceptor tyrosine activation motif (ITAM) in the cytoplasmic tail that binds to Fyn tyrosine kinase, and the ITAM may also interact with Lyn, Syk, and ZAP-70 kinases found in lymphocytes [58,59], although there is no evidence that lymphocytes are susceptible to hantaviruses. The ITAM may also promote polyubiquitination of the Gn polypeptide to facilitate its degradation [60]; however, it is unclear how it impacts the host response to infection. Presumably, the ITAM interferes with the antiviral response of an infected cell since the motif is cytoplasmic. The Gn protein may also alter the RIG-I pathway that leads to IRF3 phosphorylation and subsequent *Ifnβ* expression [61]. 

**Table 2:** Hantavirus proteins that may have immunomodulatory activities.

Viral Protein	Putative Effect
Gn ITAM	May promote ubiquitination of Gn
	Interaction with Fyn, Lyk, Syk and ZAP-70 kinases
	Inhibition of RIG-I and TBK1 pathways and IRF3 signaling
N	Interference with TNF-mediated NF-κB nuclear translocation
	Inhibition of STAT1 phosphorylation
	Inhibition of CTL-mediated apoptosis (granzyme B and caspase 3)
	Inhibition of TBK1 activation
NSs	Inhibition of *Ifnβ* expression, NF-κB and IRF-3 activities

The nucleocapsid may also antagonize the expression of *Ifnβ* by binding to importin-α and interfering with NF-κB nuclear transport, which is required for *Ifnβ* expression [62,63,64]. Additionally, both caspase 3 and granzyme B are targets of the nucleocapsid of some hantaviruses [65] and both are essential components of CTL-mediated apoptosis. The lack of damage to the endothelium of infected rodent reservoirs suggests this may be an important mechanism of preventing viral clearance. The nucleocapsid of ANDV, but not other hantaviruses, also inhibits autophosphorylation and activation of TBK1, an enzyme that activates IRF3 and NF-κB and induction of type I interferon gene expression [66]. 

Recently, putative nonstructural NSs sequences have been identified in some hantaviruses [3,67]. This sequence is in an alternative reading frame of the nucleocapsid transcript. In other bunyaviruses, NSs has anti-interferon activity [68,69,70]; however, its role in hantavirus infections is less well characterized.

Importantly, these studies have been conducted with cells from nonreservoir hosts where they, presumably, are not optimized for manipulating the immune response in a manner that benefits the virus but without host pathology. Future studies should examine the roles of these proteins in cells from reservoir hosts.

## 5. Immune Responses of Rodent Reservoirs

**Figure 1:**
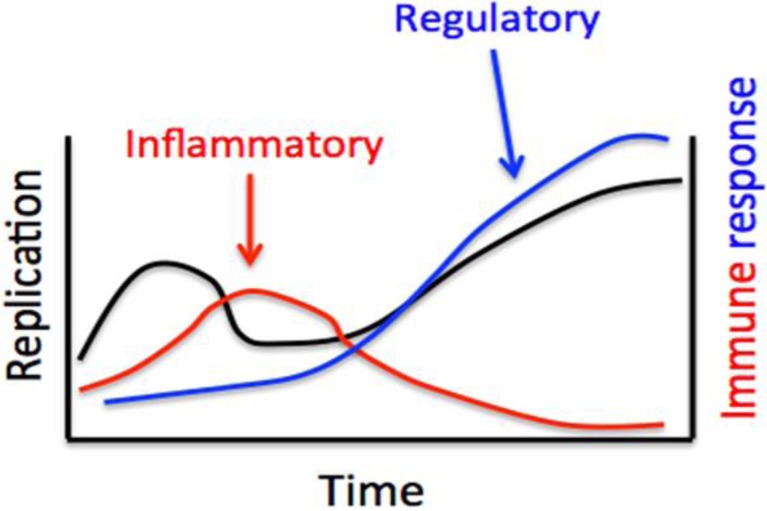
Model of the immune response of deer mice infected with Sin Nombre virus (SNV). During acute infection, SNV elicits a modest inflammatory response that initially limits, but does not clear, virus. Within a few weeks, the response transitions to a regulatory response that may allow episodic recrudescence of virus that can be shed.

The presence of high-titer IgG antibodies during hantavirus infections of reservoir hosts indicates both T cell and B cell responses occur because T cells induce class switching and affinity maturation of antibodies produced by antigen-specific B cells. In experimental infections of rats with SEOV and deer mice with SNV, early infection results in subtle inflammatory signatures, but a regulatory T cell (Treg) response predominates at persistence (Figure 1) [20,54]. Treg responses are critical for suppressing inflammation [71,72]; however, inflammation is a prominent feature of hantavirus disease in humans [73] and hamsters [55,74]. In other viral diseases, the occurrence of a Treg response is associated with persistent infection because these cells, while suppressing inflammation, also prevent virus clearance [75,76]. For reservoirs of hantaviruses, the Treg response may limit inflammatory immunopathology to an otherwise innocuous infection, but it may also impair virus clearance. How this relationship evolved is unknown, but considering the presence of hantavirus proteins with immunomodulatory activities, it suggests the viruses may manipulate the host response to favor persistence; a Treg response may prevent sterilizing immunity, thus allow virus to remain in a population. It may also explain the ecoimmunology and ecology of hantavirus infections of reservoir hosts, thus studies assessing the energetics of inflammatory and anti-inflammatory immune responses should be performed.

Much of the work examining reservoir responses to hantaviruses has been conducted in rats infected with SEOV and deer mice infected with SNV or ANDV. Using cDNA arrays, Klein *et al.* [77] identified nearly 2000 genes that were differentially expressed in male and female rats infected with SEOV. Many immune-associated transcription factors, proinflammatory, antiviral, T cell and Ig family member genes were significantly higher in females, which may reduce transmission from females.

In deer mice infected with SNV, early expression signatures of a mixed Th1/Th2/Treg response were present in virus-specific CD4^+^ T cells, including *Ifnγ*, *Il4*, *Il5* and *Tgfβ*, before transitioning to a Treg-like response at persistence [54]. The expression of immune response genes differed in the spleen, where signatures of inflammation occurred within two days, peaking by days 10 to 15 before subsiding, and lungs, where little immune gene expression occurred [13]. Some deer mice produced nucleocapsid-specific IgG by day 10 while others had IgG around day 20 or later, and neutralizing antibody was not detected until after 3 weeks.

In deer mice infected with SNV, early expression signatures of a mixed Th1/Th2/Treg response were present in virus-specific CD4^+^ T cells, including *Ifnγ*, *Il4*, *Il5* and *Tgfβ*, before transitioning to a Treg-like response at persistence [54]. The expression of immune response genes differed in the spleen, where signatures of inflammation occurred within two days, peaking by days 10 to 15 before subsiding, and lungs, where little immune gene expression occurred [13]. Some deer mice produced nucleocapsid-specific IgG by day 10 while others had IgG around day 20 or later, and neutralizing antibody was not detected until after 3 weeks.

Assessment of cytotoxic T cell responses of reservoir hosts has not been reported. Most assays that assess CTL functions require susceptible syngeneic target cells, which have been difficult to obtain with reservoir hosts. Susceptible primary cell lines from reservoir hosts have been produced [37,78], but these are typically obtained from embryonic fibroblasts, thus matching of MHC alleles for use in CTL assays is difficult.

Many zoonotic viruses antagonize the innate immune response in human cells, and their pathogenic potential often correlates with their abilities to inhibit the innate response *in vitro* ([79,80,81,82] for review). Pathogenic hantaviruses inhibit antiviral responses despite high levels of replication, whereas nonpathogenic viruses are efficiently recognized and elicit innate responses that limit replication [83,84]. This antagonistic capacity must have evolved in the reservoir hosts of these hantaviruses because humans are dead-end hosts. To date, few studies have addressed the interactions between hantaviruses and their rodent hosts *in vitro*. This is partially due to the unavailability of cell lines, and the reagents and techniques to generate primary cell cultures from the various reservoirs of hantaviruses. The Norway rat/SEOV system is the most tractable for studying virus/reservoir interactions. Inoculation of rat lung-derived endothelial cells with SEOV resulted in virus replication, but little or no induction of cytokines or chemokines, suggesting SEOV can efficiently antagonize antiviral responses [21]. Despite this, endothelial cells increased their expression of the protein PD-L1, which correlated with the ability of these cells to induce Treg cell activity. In addition, antigen presenting cells isolated from Norway rats and infected with SEOV *in vitro* were resistant to stimulation, suggesting that virus infection inhibits the normal signaling activities of these cells [85]. Thus antagonism of the innate immune response likely allows for viral replication, while at the same time promotes an anti-inflammatory response that limits immunopathology. 

Similar studies have been performed using bank vole cells infected with PUUV [78]. Embryonic fibroblasts inoculated with PUUV did not express increased amounts of *Ifnβ* or *Mx2*, although non-related viruses were able to induce up-regulation of these genes. This suggests, as with SEOV, PUUV efficiently antagonizes host innate responses in its natural reservoir. 

## 6. Syrian Hamster Models of Hantavirus Disease

The Syrian golden hamster develops an HCPS-like disease when infected with ANDV or MAPV [22,23]. Hamsters inoculated with ANDV mount an inflammatory response, as measured by elevated mRNA encoding pro-inflammatory mediators, prior to succumbing to the disease [55]. Infection also results in the activation of the adaptive immune response, characterized by antigen-specific proliferation of T cells and the generation of virus-specific antibodies [74,86]. In contrast, SNV, which is highly pathogenic in humans, replicates in hamsters, but does not cause disease and is cleared by the immune response [87]. Passaging of SNV in hamsters results in a virus that is able to replicate efficiently and cause a persistent infection similar to what is seen in the rodent reservoir, yet still does not cause disease [88]. Examination of immune responses elicited by ANDV (pathogenic in hamsters) and SNV (non-pathogenic in hamsters) showed that passaged SNV evoked a stronger adaptive immune response than did ANDV; however, ANDV infection induced a much stronger innate immune response at late time points, despite both viruses replicating to similar levels. Depletion of T cells did not alter the outcome of infection [74,86]; thus, these data suggest that, at least in the hamster model, the activation of the T cell-mediated immune response is not responsible for immunopathogenesis, and perhaps the innate immune response, either elicited from infected endothelial cells, macrophages and/or neutrophils, might contribute to disease.

## 7. Future Directions

Infectious diseases and the immune response are complex processes that are challenging to study, even with substantial reagent resources and mature methodologies. Many difficulties exist for studying hantavirus infections of their reservoir hosts, including the lack of molecular and immunological reagents, methods for experimental investigation, and that few reservoir species have been colonized for laboratory use. In addition, pathogenic hantaviruses require BSL-3 and/or ABSL-4 containment, which presents logistical hurdles for examining the reservoir host/hantavirus relationship. Despite these limitations, novel instrumentation, particularly those for transcriptome profiling (e.g., RNA-Seq) and metabolomics, and development of molecular and cellular methods, provide an opportunity to rapidly develop the tools necessary for examining reservoir host responses using a systems biology approach. The key feature of these new technologies is that they are *species-independent* in the data they generate, but they require significant computational and bioinformatics resources. 

### 7.1. RNA-Seq

Infection triggers a cascading host response that is highly orchestrated by the vertebrate immune system. Many genes are modulated (expressed or repressed) during the course of infection and identification of mRNA and noncoding RNAs can be used to identify the mechanisms that control, or fail to control, disease. Moreover, some infectious diseases, including hantavirus disease, have substantial immunopathologic components. The quantitative assessment of the *transcriptional landscape* (patterns of gene expression) can be used to profile the host responses in infected and uninfected animals of the same species, or a disease model species to reservoir host species to identify mechanisms of susceptibility or resistance. RNA-Seq is one such method for profiling transcriptional landscapes [89]. 

The depth of coverage and costs of RNA-Seq have improved dramatically in the last few years, and it is likely to become less expensive. However, the computational resources necessary for using RNA-Seq for studying host responses is substantial, often requiring hundreds of gigabytes of RAM and multicore, multiprocessor systems typically found in servers running a Linux operating system [90]. This depth is often necessary to detect RNAs that occur in extremely low abundance because their proteins are highly potent (e.g., cytokines). Even then, it is possible that differentially expressed genes may not be detected and other, more sensitive methods, such as real-time PCR, may be required to validate pathway signatures. Despite these requirements, bioinformatics tools for RNA-Seq are now widely available, many of which are free. A typical first step of differential gene expression profiling is the *de novo* assembly of all RNA-Seq samples from an experiment, which represents the totality of expressed genes from the experiment. There are several *de novo* assemblers available, including the Trinity suite [90] and Oases [91]. Each of these packages has advantages and disadvantages, thus it is important to understand how each performs assemblies, particularly isoforms that may have specific activities. Included in the Trinity package is RSEM [92] that estimates transcript abundances, including isoforms, in experimental samples by counting reads from replicates against the *de novo* assembly. An important feature of RSEM is that it does not require an annotated genome; it determines the abundance of transcripts from the unannotated assembly, identifies differentially expressed transcripts, and provides a 95% credibility interval for each gene. Additional tools, such as DESeq and edgeR [93,94] provide statistical evaluation of differential gene expression between samples and provide quantitative (higher/lower) and qualitative (on/off) data. The differentially expressed transcripts are subsequently identified by other means (e.g., BLAST) and can then be mapped to pathways (such as Reactome or KEGG) [95,96] to visualize [97] where viruses may influence the host response and identify mechanistic targets of hantaviruses.

### 7.2. MicroRNA

In recent years, microRNAs (miRNA) have been identified that are important regulators of antiviral responses. Activation of TLR/RIG-I pathways leads to the expression of several miRNAs [98] that likely are important in lymphocyte functions [99]. Because hantavirus Gn targets RIG-I [61], it is possible that these miRNAs are dysregulated, which could provide the virus an advantage over the host cell response. While miRNA expression has been evaluated in hantavirus-infected human endothelial cells, epithelial cells and macrophages [100,101], no work has been conducted to examine the role of miRNAs in reservoir host cells infected with hantaviruses. Considering the importance of miRNA in host responses, it is likely they play an instrumental role in the immunological events leading to persistent infection of the reservoir hosts. The use of RNA-Seq can identify global miRNA expression [102] and clarify their regulatory roles in infected reservoirs.

### 7.3. Metabolomics

Metabolic products can provide substantial information about the interactions of viruses and infected host cells, and the how immune system responds to infection [103,104]. The field of metabolomics is young but potentially informative for understanding hantavirus/reservoir host interactions. Many viruses metabolically remodel the host cell to optimize infection. Because metabolic products (e.g., carbohydrates, lipids, prostaglandins, *etc.*) are identical or highly similar between vertebrate species, this approach may help identify which enzymes, by virtue of their metabolic products, may be targeted by hantaviruses. While there are no reports of metabolic assessment of hantavirus infections, Rift Valley Fever virus (RVFV) modulates the activity of adenosine 5’ monophosphate-activated protein kinase (AMPK) in infected cells. This enzyme is a regulator of several metabolic pathways, including enhancement of catabolic pathways such as autophagy and ATP production, but it represses anabolic pathways, such as lipid biosynthesis. Infection of cells by several viruses, including RVFV, results in activation of AMPK and restriction of viral replication, suggesting an antiviral role for this enzyme [105]. Other studies have revealed metabolic pathway targeting by viruses [106,107,108], thus efforts to examine how hantaviruses may manipulate the metabolomes of infected cells could lead to the identification of therapeutic targets for treating hantavirus disease.

### 7.4. Cellular Immunology

The use of monoclonal antibodies has been particularly challenging for hantavirus/reservoir research. Identification of cell surface markers could shed light on what cells respond during hantavirus infection of reservoir hosts. While cell surface antigens tend to be more divergent, intracellular proteins, such as antiviral proteins, tend to be more conserved, particularly phosphoepitopes. Thus, it is likely that many available antibodies to house mouse (*Mus musculus*) or Norway rat antiviral proteins will be cross-reactive with orthologs from hantavirus rodent reservoir species. Without information as to which proteins may be of interest, screening of antibodies is a daunting and expensive task since most likely will not be informative. However, combined with RNA-Seq and metabolomic data, it is likely that target proteins and pathways can be identified so that investigators can focus their efforts and resources. In addition, software tools can help predict whether antibodies may be cross-reactive to proteins from reservoir hosts [109]. 

The use of cytokines or neutralizing cytokine antibodies to perturb the host response during infection has identified many mechanisms that contribute to susceptibility or resistance. For example, administration of IFNγ to laboratory mice facilitates clearance of LCMV, whereas antibody that neutralizes IFNγ impairs CTL responses and clearance, thus leading to persistent infection [110,111]. Depletion of immune cell subsets with antibodies can also determine the roles of those cells during infection. This approach revealed a critical role for CD4^+^ T cells for sustaining CTL responses to LCMV [112]. Other than the Norway rat, an array of cytokines and antibodies for experimental manipulation of the host response of hantavirus reservoirs is substantially limited. Thus, it is difficult to determine the mechanisms controlling the host response of reservoirs. Some cytokines are broadly cross-reactive and can be used for studying reservoir responses. Recombinant house mouse GM-CSF and human IL-2 stimulate deer mouse cells [113], and it is likely that other commercially-available cytokines can also be used. Identification of cross-reactive cytokines should be a high priority of the hantavirus community. With genome and transcriptome sequencing, many cytokine genes can be rapidly cloned into expression vectors that are codon-optimized for the expression system of choice using *de novo* synthesis services (e.g., GeneArt, Life Technologies). Moreover, some antibodies may be cross-reactive with reservoir species’ orthologs [114]. Anti-mouse CD4 (clone GK1.5) and anti-rat CD8β (clone 341) antibodies can be used to deplete CD4^+^ and CD8^+^ T cells from hamsters, respectively [74,86], and they may also be useful for reservoir host studies to examine the roles of these cells.

Finally, it is difficult to assess CTL activity in reservoir hosts. Most colonies are established with wild rodents that are highly polymorphic. This limits use of traditional CTL assays that require MHC class I-matched target cells. The generation of highly inbred strains is challenging and may result in alleles that do not represent the natural biology of hantavirus infection, thus it may not be desirable to generate highly inbred rodents. It is possible to establish MHC homozygotes with controlled breeding and screening of littermates for the same haplotypes. Even then, the generation of susceptible cell lines can be problematic. While endothelial cell lines can be generated with retroviral transformation, it is possible the cells may have activated antiviral pathways that could alter *in vitro* CTL responses. The use of growth factors for expanding endothelial cells in culture may be more attractive. Until methods are established for generating syngeneic, susceptible target cells, assessment of CTL responses in reservoir hosts will be difficult. 

## 8. Conclusions

The immunological relationships between hantaviruses and their rodent reservoir hosts are complex. Infection typically leads to disseminated infection within a few days but without conspicuous signs of disease. Expression of immune genes can be detected in as little as a few days that suggests innate and adaptive immune activation, but the magnitude of expression is substantially less than in hantavirus pathology models. The lack of reagents and methodologies for studying hantavirus reservoirs, most of which are not model organisms, presents a significant challenge. However, new technologies have recently emerged that are cost effective and species-independent, but which generate large amounts of data that require substantial computational and bioinformatics support for data reduction. With these tools, it should be possible to accelerate research to understand the relationships of hantaviruses and their reservoir hosts.
